# Prevalence and Associated Factors of Cryptococcal Antigenemia in HIV-Infected Patients with CD4 < 200 Cells/µL in São Paulo, Brazil: A Bayesian Analysis

**DOI:** 10.3390/jof8121284

**Published:** 2022-12-08

**Authors:** Evanthia Vetos Mimicos, Victor Fossaluza, Camila de Melo Picone, Camila Caroline de Sena, Hélio Rodrigues Gomes, Carolina dos Santos Lázari, Fernanda Ferreira da Silva, Erika Shimoda Nakanishi, Isabelle Vichr Nisida, Angela Carvalho Freitas, Ronaldo Borges Gryschek, Eduardo Ronner Lagonegro, Márcia Lazéra, Maria Aparecida Shikanai-Yasuda

**Affiliations:** 1 Division of Infectious and Parasitic Diseases, Hospital das Clínicas, Faculdade de Medicina, University of São Paulo, Av. Enéias C. Aguiar, 255, São Paulo 05403-000, Brazil; 2 Instituto de Matemática e Estatística, University of São Paulo, São Paulo 05508-090, Brazil; 3 Laboratory of Investigation in Neurology (LIM 15), Hospital das Clínicas, Faculdade de Medicina, Universidade de Sao Paulo, São Paulo 01246-903, Brazil; 4 Centro de Referência e Treinamento DST/Aids, State São Paulo Health Secretary, São Paulo 04121-000, Brazil; 5 Laboratory of Medical Investigation in Immunology (LIM-48), Hospital das Clinicas HCFMUSP, Faculdade de Medicina, Universidade de Sao Paulo, São Paulo 05403-000, Brazil; 6 Departament of Infectious Diseases, Faculdade de Medicina FMUSP, Universidade de Sao Paulo, São Paulo 05403-000, Brazil; 7 National Institute of Infectious Disease Evandro Chagas (INI), Fiocruz, Rio de Janeiro 21040-360, Brazil

**Keywords:** *Cryptococcus*, lateral flow assay, cryptococcal antigenemia, cryptococcal meningitis, AIDS, CD4 < 200 cells/µL

## Abstract

Cryptococcosis is a severe life-threatening disease and a major cause of mortality in people with advanced AIDS and CD4 ≤ 100 cells/µL. Considering the knowledge gap regarding the benefits of routine application of antigenemia tests in HIV-infected patients with 100–200 CD4 cells/µL for the prevention of cryptococcal meningitis (CM), we aimed to evaluate the prevalence of positive antigenemia through lateral flow assay (LFA) and associated factors in HIV-infected patients with CD4 < 200 cells/µL. Our findings of 3.49% of positive LFA (LFA+) patients with CD4 < 100 cells/µL and 2.24% with CD4 between 100–200 cells/µL have been included in a Bayesian analysis with 12 other studies containing similar samples worldwide. This analysis showed a proportion of 3.6% LFA+ patients (95% credible interval-Ci [2.5–5.7%]) with CD4 < 100 cells/µL and 1.1% (95%Ci [0.5–4.3%]) with CD4 between 100–200 cells/µL, without statistical difference between these groups. The difference between mortality rates in LFA+ and negative LFA groups was e = 0.05013. Cryptococcoma and CM were observed in the LFA+ group with 100–200 and <100 CD4 cells/µL, respectively. Considering the benefits of antifungal therapy for LFA+ patients, our data reinforced the recommendation to apply LFA as a routine test in patients with 100–200 CD4 cells/µL aiming to expand cost-effectiveness studies in this group.

## 1. Introduction

Cryptococcosis is a severe life-threatening fungal disease caused by *Cryptococcus neoformans* and *Cryptococcus gattii* that affects immunocompetent and immunosuppressed individuals, especially patients with acquired immunodeficiency syndrome. This mycosis accounts for 15% of all causes of death in people with advanced HIV disease not receiving antiretroviral therapy (ART) and CD4 ≤ 100/µL in 2014 [1], and for 19% mortality in those with CD4 < 200 cells/µL in 2020 [2]. In 2020, 179,000 cases (IQR 133,000–219,000) of cryptococcal disease were estimated in people with HIV and CD4 < 200 cells/µL, accounting for 19% (13–24%) of AIDS-related mortality [2]. 

Although AIDS-related deaths declined by 52% from 2010–2021 [3], mortality attributed to cryptococcosis is prominent among the causes of death in AIDS patients, mainly because of the delay in diagnosis and incidence in underdeveloped countries [4,5,6,7]. 

Meningitis is the most common cause of cryptococcal disease in HIV infection. There is an estimated annual global median annual incidence in the pre-ART period of 957,900 cryptococcal meningitis (CM) cases (range, 371,700–1,554,000) and 54,440 in Latin America (range, 10,900–97,900) [8]. These numbers decline in the post-ART period [1] and were estimated at 223,100 (CI 95% 150,600–282,400) in 2014 and 152,000 (IQR 111,000–185,000) in 2020, with 12,000 (IQR 9000–14,000) in Latin America [2]. 

One-year CM mortality was estimated at 70% (95% CI 59–81%) in underdeveloped countries, whereas it was 40% in middle-income (95% CI 34–46%) and 20–30% in high-income countries [1]. High mortality rates were reported in South American countries, ranging from 18–67% [9,10], and in Brazil from 47–61%, during 2000–2017 [11,12,13]. 

Aiming to interfere with the CM incidence, morbidity, and mortality, the WHO strongly recommends the routine application of antigen detection tests for the diagnosis of cryptococcal infection in asymptomatic HIV-positive patients with CD4 levels < 100/µL in regions with prevalence ≥ 3% [14,15]. Moreover, it has been reported to be cost-effective, even with a prevalence as low as 0.6% [16] or 1.4% [17]. Tests are also conditionally recommended in patients with CD4 < 200/µL [4] (WHO 2022), but it is still a challenge to demonstrate the cost–benefit of the routine application in patients with CD4 counts between 100 and 200 cells/µL [18,19,20,21]. 

Recently, an easy-to-perform fast (10 min) point-of-care immunochromatographic assay (lateral flow assay—LFA) using either blood, sera, or plasma along with cerebrospinal fluid (CSF) for diagnosing CM has been reported. Its sensitivity is 100% (CI 95% 98–100%) and specificity 99% (CI 95% 97–99.4%) [22]. 

Different prevalences of antigenemia have been recorded by lateral flow assay in different regions of the world, ranging from 1.69% to 16.66% [17,19,20,21,23,24,25,26,27,28,29,30,31]; even within Brazil itself, this prevalence varies from 1.3–11.66% [23,27,28,29]. 

As recommended by WHO [14,15], patients with negative symptoms and positive antigenemia can be prescribed preemptive antifungal therapy that targets cryptococcal disease. This strategy is largely cost-effective compared to inpatient treatment of CM, considering both the CRAG-latex [16,32] and LFA [17,19] diagnostic tests. In addition to the benefit of being a point-of-care test, in Brazil, the cost–benefit of LFA is even higher than the CRAG-latex in people living with HIV/AIDS (PLHA) with CD4 ≤ 200 cells/µL [33].

Finally, considering a significant worldwide knowledge gap about the consequences of cryptococcal antigen screening in HIV-infected patients with CD4 < 200 cells/µL, the present study aims to estimate the prevalence of positive cryptococcal antigenemia, as well as the difference in prevalence between CD4 < 100 cells/µL and 100–199 cells/µL groups. We also aim to analyze possible factors associated with positive antigenemia in PLHA with CD4 < 200 cells/µL.

## 2. Materials and Methods

We conducted a cohort study, carried out from December 2014 to February 2018 at two HIV care services in the city of São Paulo, Brazil. The first at Hospital das Clínicas da Faculdade de Medicina da USP (HCFMUSP) included inpatients and outpatients of the Infectious and Parasitic Diseases Division (Serviço de Extensão ao Atendimento de pacientes HIV/Aids) and patients from an emergency room of this hospital. The second is the Centro de Referência em HIV/Aids/Doenças Sexualmente Transmitidas of the Health Department of the State of São Paulo, Brazil (CRTA-SES-SP).

### 2.1. Patients and Follow Up

The inclusion criteria were having a confirmed HIV infection by testing positive for HIV1/HIV2 antigens through ELISA and immunoblot, being ≥18 years old, and CD4 < 200 cells/µL.

The exclusion criteria were being younger than 18 years of age, having a history of previous cryptococcosis, having confirmed cryptococcosis at the time of enrollment, or having a consistent suspicion of cryptococcosis by clinician judgment.

The sample size was estimated as 230 HIV-infected patients, based on an α error of 0.05 and an β error of 0.1 with a power test of 90%, with the possibility of including more participants based on test availability. 

The selection of patients was done using a convenience sample, with a balanced distribution between sites and CD4 cells stratum (<100 cells/ µL and between 100 and 200 cells/µL).

For 244 patients, whole blood (5 mL) was collected after enrollment and, for 33 patients, samples previously collected and stored at −70 °C were employed for LFA analyses.

Clinical and laboratory data were collected during enrollment or from medical records (general symptoms, duration of antiretroviral use, duration of HIV diagnosis, opportunistic diseases, previous use of antifungals, and CD4 cell count and HIV viral load (VL) results).

Medical and laboratory records were reviewed at 3, 6, 9, and 12 months and in 2021 and 2022 during the follow-up of LFA+ patients to check on the evolution of preemptive antifungal therapy, the incidence of cryptococcal disease, adherence to ART, CD4, and VL, and rate of mortality; survival and death data were checked in February 2021 and February 2022. Regarding negative LFA patients, death records were verified 12 months after inclusion.

### 2.2. Lateral Flow Assay

All samples were stored at −70 °C.

The lateral flow assay (LFA) was performed using a point-of-care serum rapid antigen test (IMMY, Immuno-Mycologics Inc. Norman, OK, USA) as per the manufacturer’s instructions.

Patients who had positive antigenemia by LFA were invited for clinical reassessment and lumbar puncture for diagnosing CM through India ink microscopy, culture, and cerebrospinal fluid LFA. When CM was confirmed, liposomal amphotericin and fluconazole were prescribed. If no cryptococcal disease was confirmed, preemptive treatment with 800 mg fluconazole for 2 weeks, 400 mg for 8–10 weeks, and a daily maintenance dose of 150–300 mg were recommended. Patients continued to be reassessed by their physicians at the clinic of origin.

Data on the evolution of CD4 count and HIV viral load were accessed using the Sistema de Controle de Exames Laboratoriais (SISCEL), a national network with CD4+/CD8+ lymphocyte counts and HIV viral load (VL) from the Secretariat of Health Surveillance, Ministry of Health, Brazil.

Mortality data were assessed by the Health Surveillance Information Center (CIVS), Coordination of Disease Control of the Health Secretariat of São Paulo State, Brazil.

### 2.3. Ethics

The project was approved by the Research Ethics Committee of Hospital das Clínicas da Faculdade de Medicina, University of São Paulo, Brazil, under protocol No. 12457. It was also approved by the Ethics Committee of CRTA/SES/SP under protocol No. 1.765.576 and registered in the National Research Ethics Committee, Conselho Nacional de Saúde, Ministry of Health, Brazil under CAAE No. 36061214.1.0000.006.

All subjects provided informed consent before enrollment and all subject identifier data were scrubbed from the database.

### 2.4. Statistical Analyses 

Considering the low frequencies of positive LFAs observed in our sample, it was understood that the Bayesian approach to data analysis would provide more robust results than traditional statistical methods. This methodology allows the use of prior data, extracted from similar previous studies, incorporating them as a landmark to correct possible bias within the findings. Other advantages of Bayesian inference can be found in [34,35] and in the references therein.

In this work, we considered data from previous articles reported up to July 2021 that studied similar populations where the LFA test of the same manufacturer had been applied to people with CD4 < 100 cells or <200 cells/µL to build the prior distributions. The articles were selected by two researchers in the Pubmed databases using the keywords: “Lateral Flow Assay” and “Cryptococcal antigenemia”, “Cryptococcal antigenemia” and “CD4”, and “Cryptococcal antigenemia” and “HIV”, secondary references, and institutional data. Articles were excluded under the following criteria: containing patients with confirmed cryptococcosis, younger than 18 years, with CD4 levels > 200 cells/µL, or only presenting CD4 levels < 100 cells/µL. Articles that did not define the method used for antigenemia analysis or that used a method other than the “Lateral Flow Assay” were also excluded.

The prior distribution was obtained by considering a mixture of densities from each study weighted by their sample sizes, called meta-analytic distribution [36,37], which allows for considering the variability between data from different studies. 

**Figure 1:**
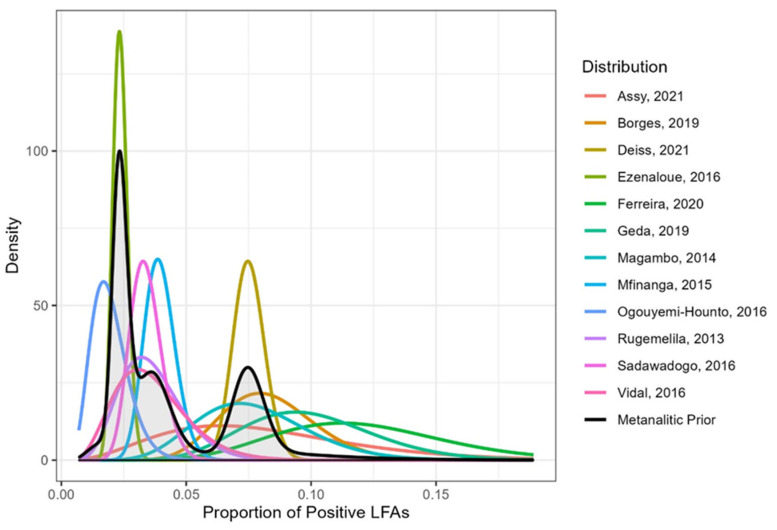
Densities related to each study and the meta-analytic prior distribution for the proportion of positive LFAs [19,20,21,23,24,25,26,27,28,29,30,31].

The studies considered were Assy et al., Brazil (*n* = 47, *x* = 3) [29], Borges et al., Brazil (*n* = 214, *x* = 17) [28], Deiss et al., Mozambique (*n* = 1795, *x* = 134) [31], Ezenaloue et al., Nigeria (*n* = 2752, *x* = 64) [26], Ferreira et al., Brazil (*n* = 89, *x* = 10) [27], Geda et al., Ethiopia (*n* = 128, *x* = 12) [30], Magambo et al., Tanzania (*n* = 140, *x* = 10) [20], Mfinanga et al., Tanzania and Zambia (*n* = 985, *x* = 38) [19], Ogouyemi-Hounto et al., Benin, (*n* = 355, *x* = 6) [25], Rugemalila et al., Tanzania (*n* = 218, *x* = 7) [21], Sadawadogo et al., Namibia (*n* = 825, *x* = 27) [24], and Vidal et al., Brazil (*n* = 163, *x* = 5) [23], where *n* is the sample size and *x* is the number of positive LFAs from each study. Figure 1 shows the densities of each study and the meta-analytic prior distribution.

To construct the prior distribution for each subgroup, all studies that presented the proportion of positive LFA results for that subgroup were considered. Hypothesis testing and group comparisons were performed using the full Bayesian significance test (FBST) [38] and the significance level adopted was 5% and 95%Ci is the 95% credible interval.

In addition, the prevalence of LFA positivity was estimated by the posterior mode, and higher posterior density credible intervals (HPD Ci) were presented. All statistical analyses were performed in R language [39] using R-Studio [40]. The graphs were generated with the ggplot2 package [41].

Table 1 shows the studies included in prior distribution according to the analyzed group, number of LFA+ cases, and sample size. 

## 3. Results

Of a total of 304 recruited patients, 27 were excluded because of either previous cryptococcosis or suspected CM, duplicate inclusions, loss of material without backup, and previous positive tests for *Cryptococcus* such as culture or histopathological analysis. Thus, 277 patients were included, specifically, 244 before sample collections, and 33 whose samples were previously collected and stored at –70 °C.

### 3.1. Prevalence of Positive Antigenemia 

Table 2 shows the results of the LFA tests in 277 patients living with HIV/AIDS according to the socio-demographic and other analyzed variables, as well as the number of positive LFA samples. The prevalence of LFA+ was 2.89% (8/277) in patients with CD4 < 200 cells/μL, 3.49% (5/143) for those with CD4 < 100 cells/μL, and 2.24% (3/134) with CD4 100–199 cells/μL.

At inclusion, more than 50% were male, and the median age was 43.97 years ± 11.5 SD (range 18–73). Most lived in the city of São Paulo (*n* = 200) and in other cities of São Paulo State (*n* = 75); two lived in other states. Most had opportunistic AIDS infections and symptoms not consistent with cryptococcosis suspicion, and 33.2% were outpatients, as shown in Table 2. Although non-nucleoside reverse transcriptase inhibitors and protease inhibitors had been prescribed; most patients were on irregular use of ART, which was confirmed by the detection of HIV RNA viral copies/µL.

### 3.2. Follow-Up of Patients with Positive LFA 

Table 3 shows the main characteristics of the eight patients who tested positive for LFA. Among five patients with a CD4 count lower than 100 cells/µL, two (P4 and P6) had confirmed CM (*Cryptococcus* identified in the culture and India ink microscopy in addition to positive LFA of CSF) and survived after being prescribed Liposomal amphotericin and fluconazole: one patient (P7) came from another hospital with a previously performed lumbar puncture, without CSF abnormalities, and received fluconazole for seven days for esophageal candidiasis but abandoned the routine outpatient care and was still alive until the end of follow-up. The remaining two patients refused to perform a lumbar puncture. Specifically, (P2) dropped out of the routine outpatient care follow-up and died of bacterial sepsis 21 months later; (P8) dropped out of the routine outpatient care and was still alive until the end of the follow-up. 

Three patients had a CD4 count between 100 and 199 cells/µL. One had pulmonary cryptococcosis (P1), confirmed by image-guided biopsy, and improved after having received fluconazole but died 2.7 months later of bacterial sepsis. Two patients refused to perform a lumbar puncture: P3 dropped out of the routine outpatient care and was still alive until the end of the follow-up without hospitalization, and P5 was asymptomatic and his physician preferred to repeat the antigenemia analysis two months later. Antigenemia was negative and the patient survived until the end of the follow-up.

Two patients died of sepsis with low CD4 cell counts and high viral loads. All six survivors had <200 CD4 cells/µL and undetectable VL (P3-P8, Table 3) at the end of the follow-up (48–78 months); in two of them, antifungal therapy for meningitis was prescribed. 

### 3.3. Prevalence of LFA Positivity by Bayesian Analysis in 13 Studies

**Figure 2:**
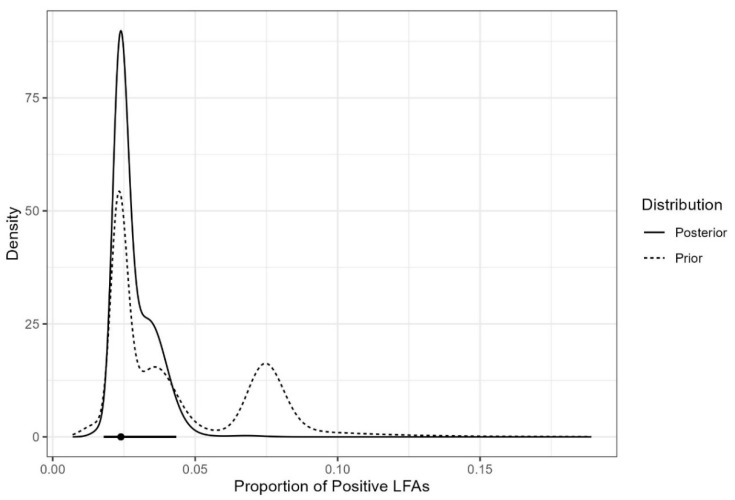
Prior and posterior densities of the proportion of positive LFAs with estimated prevalence and HPD credible interval.

**Figure 3:**
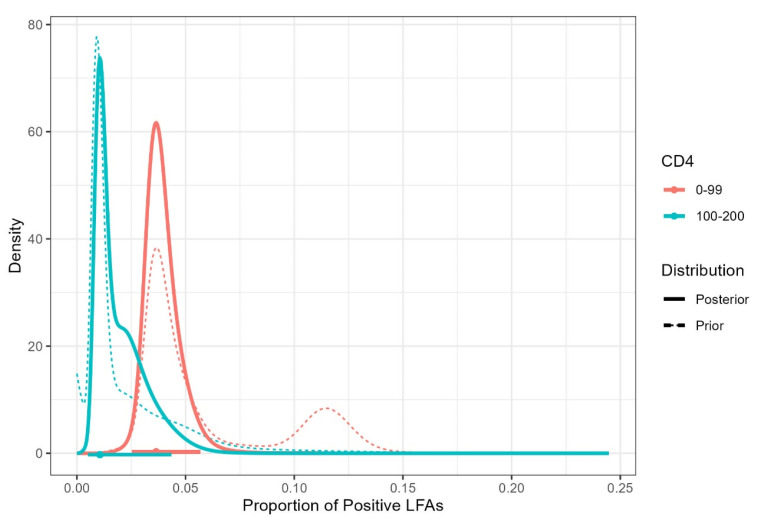
Prior and posterior densities of the proportion of positive LFAs with estimated prevalences and HPD credible intervals for each CD4 group.

The results of the Bayesian analysis are presented below (Figure 2 and Figure 3, and Table 4 and Table 5).

The graph of posterior density considering the meta-analytic prior is presented in Figure 2. The estimate of the proportion of LFA positivity is 0.024 and the 95% credible interval is [95% Ci 0.018; 0.043]. Using FBST to test the hypothesis that the prevalence of LFA positivity is greater than 0.03, we obtain an evidence value equal to 0.365, indicating that this hypothesis should not be rejected. Next, the CD4 groups are compared. Assy et al. (2021) [29] do not present data divided by the same CD4 categories and, therefore, this work was not considered in the construction of priors for this comparison. All other works included in the overall analysis were considered here. The estimate of the proportion of positive LFAs in the 0–99 cells/µL CD4 group is 0.036 and the 95% credible interval is [95% Ci 0.025; 0.057]. In the 100–200 cells/µL CD4 group, the estimate is 0.011 and the 95% credible interval is [95% Ci 0.005; 0.043].

Although the group with CD4 0–99 cells/µL has a slightly higher proportion of positive LFAs, there is a large overlap of credibility intervals and the FBST to test equality between groups provides an e-value equal to 0.307, indicating that this hypothesis should not be rejected.

Table 4 and Table 5 below show the studies considered in the construction of prior distributions for each comparison performed.

There is no difference in antigenemia prevalence between PLHA with CD4 lower than 100 cells/µL and 100–200 cells/µL (e = 0.307). Regarding the distribution of all other variables—age, hospitalization, regular ART, viral load, opportunistic infections, symptoms, and antifungal therapy—there are no differences between antigenemia-positive or -negative patients (e > 0.05). 

### 3.4. Mortality Rates in Positive LFA and Negative LFA Groups: Bayesian Analysis in Three Studies

**Figure 4:**
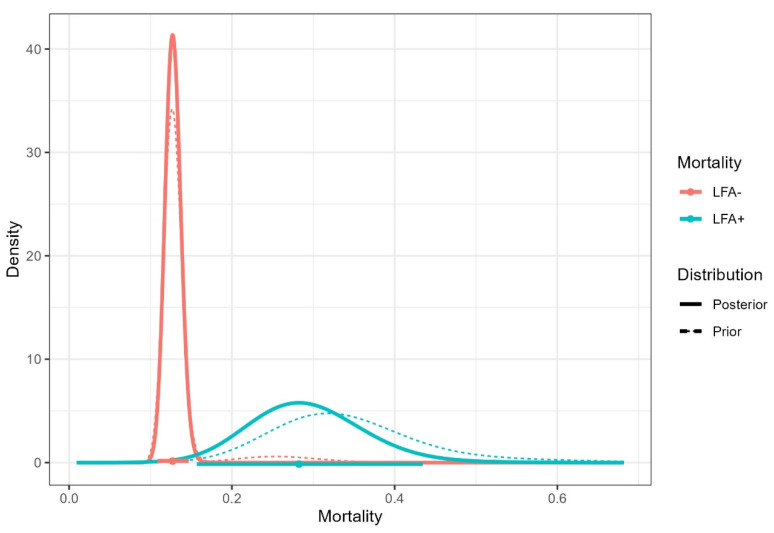
Prior and posterior densities of the mortality proportion with estimated prevalences and HPD credible intervals for positive and negative LFA groups.

The overall one-year mortality rate of all the subjects enrolled in our study was 12.8% (33/257). In the negative LFA group, it was 12.9% (32/249), and 12.5% (1/8) in the LFA+ group (Table 1). To estimate the difference between overall one-year mortality rates in LFA+ and negative LFA groups, we applied the Bayesian analysis considering the prior distribution of two other similar studies: Mfinanga et al., 2015 [19]: 12/38 deaths in the positive LFA and 120/947 in the negative LFA group; and Ferreira et al., 2020 [27]: 4/10 and 19/71, respectively. The result is shown below (Table 6 and Figure 4).

Among the 269 negative LFA patients, one patient with 48 CD4 cells/µL and high viral load had CM 10 months after the original screening test and died.

## 4. Discussion 

The comparative analysis of the prevalence found by the Bayesian analysis in patients with CD4 ≤ 100 cells/µL was like the one observed in patients with CD4 between 100 and 200 cells/µL, although in disagreement with previous meta-analyses [42,43]. The importance of this similarity between both cell ranges is reinforced by the finding of cryptococcosis in LFA+ patients in this last stratum in several studies [27,31]. In addition, after testing the hypothesis that the prevalence of LFA positivity is greater than 3% in participants with CD4 < 200 cells/µL, as proposed by WHO for routine *Cryptococcus* antigen screening in such HIV-infected patients [14,15], our evidence indicates that this hypothesis should not be rejected. This finding agrees with the global estimate in 2020 of 4.4% (95% CI 1.6–7.4%) [2] in a large size sample including several methods of antigenemia analyses. Considering the higher quality of evidence provided by Bayesian analysis, we consider that our results are crucial to support decision-making [34,35].

Among our 277 patients with CD4 < 200 cells/μL, without suspected or confirmed cryptococcal disease, a prevalence of 2.89% of LFA positivity was observed, similar to that previously described in asymptomatic patients by another reference institution in São Paulo (3.1%) [23]; both were lower than those described in Pará (6.38%), Goiás (7.94%), and Rio de Janeiro (RJ) (11.24%) [27,28,29]. Considering only patients with asymptomatic antigenemia in Goiás and RJ, the prevalences were 4.8% and 5.3%, respectively [27,28]. In Pará, Brazil, in an asymptomatic population with CD4 ≤ 200 cells/μL, 8.3% of positive antigenemia by CRAG-latex was described in 2013 [44]. These differences are probably related to geographic influences, the structure of local health services, programmatic actions to promote adherence to ART adopted by regional and local governments, and local environment reservoirs of the infectious agent. They could also be related to different study designs, such as the units of patients’ recruitment, the proportion of hospitalized patients, and the exclusion criteria of highly suspected cases of cryptococcosis. In RJ, the prevalence was the highest in Brazil within this series, and the high frequency of CM is justified by the screening in the emergency department (8) where a considerable proportion of advanced HIV disease could have been found [27]. 

For comparison with estimates from other regions of the world, positive LFA prevalence in patients with ≤200 cells/μL ranged from 1.69% in Cotonou, Benin to around 7% in Tanzania, Ethiopia, and Mozambique, to values higher than 10% in the north of Brazil [19,20,21,22,23,24,25,26,27,28,29,30,31]. Two previous meta-analyses among patients from several continents with CD4 < 200 cells/μL from 1997 to 2017 and from 1989 to 2021 found antigenemia prevalences of 5% (95% CI 2–7%) [43] and 4.4% (95% CI 1.6–7.4%) in the same CD4 group [2]. So, our study maintains the prevalence results at the expected range of previous analyses registered in regions of lower socioeconomic development, reinforcing the burden of cryptococcosis, and the importance of surveillance regarding early diagnosis of severe forms, particularly CM, through antigenemia screening and timely introduction of antifungal therapy.

While searching for more accurate data on CD4 counts regarding cryptococcal antigenemia screening to guide preemptive therapy, we found a prevalence of positive antigenemia of 3.6% (95% Ci 2.5–5.7%) among patients with CD4 ≤ 100 cells/µL and 1.11% (95% Ci 0.5–4.3%) among patients with CD4 between 100 and 199 cells/µL. This analysis showed a large overlap of credibility intervals, accepting the hypothesis of a prevalence greater than 3% in the extract of CD4 between 100 and 200 cells/µL. Comparing our data with three previous meta-analyses carried out with various methods of antigenemia analysis in different periods and regions of the world [2,42,43] we found lower estimates despite including previous high prevalence data from some regions. The first meta-analysis reported an antigenemia prevalence of 6.5% (95% CI 5.7–7.3%) in patients with CD4 ≤ 100 cells/µL and 2.0% (95% CI 1.2–2.7%) with 101 to 200 cells/µL (studies reported from 2003–2014) [42]. In the other cited systematic review, the estimated prevalences were 6% (95% CI 2.0 = 11.0%) for CD4 ≤ 100 cells/μL and 2.0% (95% CI 1.0- 3.0%) for CD4 between 100 and 200 cells/μL (studies reported from 1997–2017) [43]. 

These lower values estimated in our work using the Bayesian analysis among patients with CD4 cells ≤ 100/μL can be attributed to the inclusion of patients already on ART because of the admittance of a more recent period (2007 to 2021) in which the LFA was the unique test included. Other differences could be attributed to the regions involved, sample sizes, different levels of adherence to ART, and the proportion of hospitalized patients, among others. 

Next, we analyzed the beneficial effects of screening for cryptococcal disease, especially CM in patients with positive antigenemia, followed by antifungal therapy. Two out of five of our patients with CD4 < 100 cells/μL of CM (P4 and P6) survived after treatment with liposomal amphotericin and fluconazole (Table 3). Additionally, one out of three patients with a CD4 count between 100 and 200 cells/μL was diagnosed with pulmonary cryptococcosis and successfully treated. No death by cryptococcosis or incidence of this disease was shown in the follow-up of eight LFA+ patients. Overall mortality rates in LFA+ patients were 25% in our work and 11.7% [28] without any death by cryptococcal disease. Over 12 months, these rates were 12.5% in our work, 31.6% [19], and 40% [27], both with deaths by cryptococcosis. Besides the reports of mortality rates by cryptococcosis of 37.9% [31] and 33.3% [27] confirming its severity, the difference in overall one-year mortality rates between positive and negative LFA groups was close to the threshold value of significance (e-value = 0.05013). This was possibly due to the timely use of antifungal preemptive therapy in LFA+ patients with confirmed cryptococcosis, the small number of LFA+ patients in our study, the severity of cryptococcosis, and the small number of prior studies [19,27]. 

Such data are supported by previous studies showing a 40% reduction in the incidence of CM and lower mortality rates in patients with positive antigenemia under antifungal therapy compared to those without therapy [42,43]. In addition, the only death by cryptococcosis in our study occurred in one negative LFA patient 10 months after the original screening, similar to previously reported [45], and is possibly not related to a false negative result because of this large interval. 

Concerning the association between CD4 counts and positive antigenemia, this study fails to find an association, in contrast to the studies in Nigeria and Cambodia, where CD4 ≤ 50 cells/µL was a predictor for positive antigenemia [46,47], and in Brazil, where CM was associated with lower counts of CD4 [27]. 

Given the criteria strongly recommended by the WHO for screening patients with CD4 < 100 cells/µL, 12% to 40% of positive LFA patients included in the present Bayesian analysis would not have been known as positives because their CD4 counts were between 100 and 200 cells/µL, a screening value that is not strongly recommended. Accordingly, 9% of infected HIV participants in two cohorts diagnosed in the first episode of cryptococcosis presented CD4 ≥ 100 cells/μL [48]. It should also be noted that in one of the regions with a high prevalence of positive antigenemia in Brazil, this estimate was higher in patients with CD4 between 100 and 200 cells/µL (9.4%) than in those with CD4 < 100 cells/µL (7.5%) [28]. Remarkably, CM occurred in 5.9–16.7% of LFA+ patients within the 100–200 cells/µL range [27,28,31] and pulmonary cryptococcosis in 12.5% (present work), with high morbidity and mortality. We recommend expanding the studies on the benefits of screening within 100 and 200 cells/µL CD4 range, aiming to further understand the impact on the incidence of CM or other cryptococcal diseases and mortality prevention in this group [26,42,43,49,50]. 

According to this recommendation, the cost-effectiveness of antigenemia screening programs associated with antifungal therapy for positive antigenemia patients and home visits for ART initiation compared to standard care was shown in a randomized work in Africa with a 28% mortality reduction in HIV-infected patients with CD4 cells < 200 cells/μL [19]. For patients with CD4 < 100 cells/μL, studies employing the CRAG-latex test have already demonstrated a 20–40% reduction in disease-associated mortality [51,52], preventing 43% of CM deaths even at a low prevalence of 1.4% [17]. In Brazil, a cost-effectiveness study based on the prevalence of 3.09% in HIV-infected patients with CD4 < 200 cells/μL showed that two cryptococcal antigen tests (CRAG-latex and LFA) are cost-effective in patients with CD4 < 200 cells/μL compared to India ink and no screening, with LFA proving superior to CRAG-latex, avoiding 15% of deaths by CM and decreasing costs by 11 million US dollars in five years [33]. Recently, the cryptococcal antigen lateral flow assay diagnostic test for the detection of *Cryptococcus* infection in PLHA was incorporated for use in the Unified Health System (SUS) by the Ministry of Health, for patients with CD4 < 200 cells/µL and for the diagnosis of CM in PLHA regardless of CD4+ cell count [53,54].

The present work has limitations. Our sample is small and restricted to two centers in São Paulo City. Additionally, despite being included in the reference units, some of the patients were treated in other services and dropped out from the follow-up, resulting in a loss of information. This occurred mainly with patients whose inclusion was done retrospectively for having previously collected and stored serum. The abandonment was partially circumvented by the recovery of tests related to HIV infection in the National Data System (Siscel). We cannot exclude the possibility that some studies might not have been included in the Bayesian analysis, but our sample is representative of several regions of low socioeconomic status in the world. Furthermore, the inclusion of studies that only used lateral flow assays may have limited the sample but met the criteria of the proposed statistical analysis.

The strengths of this work are represented by the Bayesian analysis in several studies carried out with LFAs around the world, reinforcing the benefits of screening among patients with CD4 between 100–200 cells/µL who have positive antigenemia and cryptococcal disease and may benefit from screening programs and preemptive antifungal therapy associated with health care for patients not yet integrated with effective antiretroviral therapy. Along with this, we confirmed that the timely use of preemptive antifungal therapy prevented deaths from severe cryptococcosis in the LFA+ group. 

In Brazil, referral centers of the Brazilian AIDS Program across the country offering ART and access to LFA screening and preemptive antifungal therapy to patients with CD4 < 200 cells/µL are expected to support a continuous and sustained intervention to ensure surveillance in the prevention of CM and cryptococcal deaths. 

Finally, the incorporation of the LFA test by the Brazilian Ministry of Health in June 2021 represents an excellent opportunity for cost-effectiveness analysis of screening and preemptive antifungal therapy in large cohorts of HIV-infected patients with CD4 between 100–200 cells/μL, aiming to establish operational and decision-making strategies to reduce the burden of cryptococcosis among us [54]. 

## 5. Conclusions

The present study showed, through Bayesian analysis, in HIV-infected patients with CD4 < 200 cells/μL screened by LFA, that the estimated prevalence for CD4 between 100 and 200 cells/μL is like that found for those with CD4 ≤ 100 cells/μL, not rejecting the hypothesis that this prevalence is greater than 3%. Furthermore, a similar analysis is shown for CD4 cells < 200 cells/μL. Based on our study, the extension of antigenemia screening to patients with CD4 between 100 and 200 cells/uL benefits 10–40% of LFA+ patients as well as 5.9% to 16.7% of patients bearing potentially fatal cryptococcal diseases. It is a challenge to understand the association between the incidence of asymptomatic CM and high blood cryptococcal titers, which could be used to provide preemptive antifungal therapy to patients with advanced HIV not undergoing ART in regions where the lumbar puncture is less accessible but can provide antifungal therapy.

Thus, the expansion of knowledge of the cost–benefit of applying screening and antifungal therapy in this CD4 stratum, combined with primary care in the administration of ART worldwide, is extremely necessary to disseminate the application of antigen testing in regions with high disease prevalence to prevent severe sequelae and deaths from cryptococcosis.

## Figures and Tables

**Table 1:** Studies included in the prior distribution for each group with the number of positive LFA cases and sample sizes for each study.

Author	Sex		Age (Years)		Hospitalization		ART		Opportunistic Disease		Viral Load RNA Copies/μL		Time Since Diagnosis		Symptoms		Mortality ^a^ n/N (%)	
	n/N (%)		n/N (%)		n/N (%)		n/N (%)		n/N (%)		n/N (%)		n/N (%)		n/N (%)			
	F	M	<40	>40	No	Yes	Regular Use	Irregular Use	No	Yes	<1,000,000	≥100,000	≤12 mo	>12 mo	No	Yes	LFA+	Negative LFA
Rugemalila, 2013	5/124 (4.03%)	2/94 (2.13%)	NA	NA	7/218 (3.21%)	NA	1/96 (1.04%)	6/122 (4.92%)	NA	NA	NA	NA	NA	NA	NA	NA	NA	NA
Magambo, 2014	6/81 (7.41%)	4/59 6.78%	NA	NA	NA	NA	NA	NA	NA	NA	NA	NA	NA	NA	NA	NA	NA	NA
Mfinanga, 2015	NA	NA	NA	NA	38/985 (3.86%)	NA	NA	NA	NA	NA	NA	NA	NA	NA	NA	NA	12/38 (31.6%)	1120/947 (12.7%)
Ezenaloue, 2016	33/1570 (2.10%)	31/1182 (2.62%)	NA	NA	64/2752 (2.33%)	NA	NA	NA	NA	NA	NA	NA	NA	NA	NA	NA	NA	NA
Ogouyemi-Hounto, 2016	3/205 (1.20%)	3/150 (2.00%)	NA	NA	6/355 (1.69%)	NA	4/289 (1.38%)	2/66 (3.03%)	NA	NA	NA	NA	NA	NA	NA	NA	NA	NA
Sadawadogo, 2016	11/374 (2.94%)	16/440 (3.64%)	NA	NA	27/825 (3.27%)	NA	NA	NA	NA	NA	NA	NA	NA	NA	NA	NA	NA	NA
Vidal, 2016	2/64 (3.13%)	3/99 (3.03%)	NA	NA	NA	5/163 (3.07%)	NA	NA	NA	NA	NA	NA	NA	NA	NA	NA	NA	NA
Borges, 2019	3/59 (5.08%)	14/155 (9.03%)	11/112 (9.82%)	6/102 (5.88%)	13/168 (7.74%)	4/46 (8.70%)	11/110 (10.00%)	6/104 (5.77%)	4/107 (3.74%)	13/107 (12.15%)	10/104 (9.62%)	7/110 (6.36%)	7/101 (6.93%)	10/113 (8.85%)	13/139 (9.35%)	4/75 (5.33%)	NA	NA
Ferreira 2020	5/28 (17.86%)	5/61 (8.20%)	NA	NA	10/89 (11.24%)	NA	NA	NA	NA	NA	NA	NA	NA	NA	NA	NA	4/10 (40.0)	19/71 (26.8%)

Note *n* = (numerator) samples + for the variable N (denominator) = total number of samples; F = female; M = male; ART—antiretroviral therapy; mo = months. ^a^ Overall one-year mortality rates in positive and negative LFA groups. NA = not available. Antifungal use: no/yes—not available.

**Table 2:** Distribution of patients according to frequencies of positive lateral flow assays and sociodemographic, clinical, and laboratory characteristics.

Group	Subgroup	N (%)	Positive LFA (N)
Region of Birth	Southeast	205 (74.0)	6
	Northeast	54 (19.5)	2
	Others	18 (6.5)	0
Race	White	187 (67.5)	4
	Afro-American	81 (29.3)	3
	Others	2 (0.7)	0
	No available	7 (2.5)	1
Age	≤40	94 (34.0)	3
	>40	183 (66.0)	5
Sex	Female	98 (35.4)	5
	Male	179 (64.6)	3
Sexual Orientation ^a^	Heterosexual	166 (66.7)	7
	Homosexual	83 (33.3)	1
Education	≤8 years	122 (44.0)	4
	>9 years	144 (52.0)	3
	Not available	11 (4.0)	1
Time Since Diagnosis ^b^	≤12 months	76 (27.8)	5
	>12 months	197 (72.2)	3
Hospitalization ^c^	No	106 (38.8)	3
	Yes	150 (61.2)	5
Symptoms	No	47 (17.0)	1
	Yes	230 (83.0)	7
Opportunist Disease ^d^	No	104 (39.2)	3
	Yes	161 (60.8)	5
CD4 (cells/μL)	0–100	143 (51.6)	5
	100–200	134 (48.4)	3
Viral Load (copies/μL)	<100.000	86 (31.0)	2
	≥100.000	191 (69.0)	6
ART	Regular Use	71 (25.6)	2
	Irregular Use	206 (74.4)	6
Antifungal Use ^e^	No	243 (88.4)	8
	Yes	32 (11.6)	0
Meningoencephalitis	No	275 (99.3)	6
	Yes	2 (0.7)	2
Mortality ^f^	No Yes	224 (87.2) 33 (12.8)	07 01

**N:** number of observations in each group; positive LFA: number of positive LFA cases observed in each group, ^a^ 28 missed information, ^b^ 4 missed information; ^c^ 21 missed information (patients selected from serum bank), ^d^ 12 missed information; ^e^ 2 missed information, ^f^ one-year overall mortality, 20 missed information.

**Table 3:** Characteristics of the eight positive LFA patients, pre-emptive antifungal therapy, antiretroviral treatment, and outcome.

Patients	Patient 1	Patient 2	Patient 3	Patient 4	Patient 5	Patient 6	Patient 7	Patient 8
Inclusion *	02/10/2015	06/16/2015	07/23/2015	10/21/2015	02/16/2017	03/30/2017	04/05/2017	10/16/2017
Hospitalization	Yes	No	No	Yes	No	Yes	Yes	Yes
Age (years)/Sex	54/Male	38/Female	60/Female	38/Female	49/Male	21/female	40/Female	54/male
Ethnicity	White	White	Afro-American	Afro-American	White	Afro-American	NA	White
State of birth	São Paulo	Pernambuco	São Paulo	São Paulo	Minas Gerais	São Paulo	Bahia	Minas Gerais
Date of HIV diagnosis	02/01/2015	01/01/1997	11/01/2014	10/01/15	09/30/2003	02/01/2017	07/01/2005	09/02/2017
Sexuality	Hetero	Hetero	Hetero	Hetero	Hetero	Hetero	NA	Bisexual
Opportunistic infections	Neurotoxo	Neurotoxo	Cachexia	Myelitis	No	KPC bacteriuria	Neurotoxo	Ocular syphilis
Symptoms at inclusion	No	No	No	Yes	No	Yes	Yes	Yes
CD4 cells/µL(inclusion)	121	77	159	95	137	13	38	73
RNA viral copies/µL (inclusion)	8,602,804	1116	<40	336	<40	364	34,505	131,527
LFA+ blood/CSF	POS/NA	POS/NA	POS/NA	POS/POS	POS/NA	POS/POS	POS/NA	POS/NA
Cryptococcosis	Pulmonary	No	No	Meningitis	No	Meningitis	No	No
Antifungal therapy	Fluconazole	refused LP abandonment	refused LP abandonment	Lipo Amp + fluconazole	refused LP, negative LFA in the blood two months later.	Lipo Amp + Fluconazole	Fluco *Candida* abandonment	abandonment
Outcome(m = months)	† sepsis 2.7 m	† sepsis 21 m	Alive 48 m	Alive 78 m	Alive 48 m	Alive 63.5 m	Alive 60 m	Alive 58 m
CD4 cells/µL **	04/06/2015 124	01/09/2017 20	06/07/2022 429	04/27/2022 377	01/20/2021 270	07/07/2022 455	05/28/2022 211	06/01/22 390
RNA viral copies/µL **	860,804	165,608	undetectable	undetectable	undetectable	undetectable	undetectable	undetectable
Last ART	3tc/tdf/efv	NA	3tc/dol	tdf/3tc/dol	tdf/3tc/efv	NA	NA	NA

* Inclusion/date when diagnosed with HIV: month/day/year, ** follow-up date as indicated. Hetero = heterosexual. Neurotoxo = neurotoxoplasmosis. KPC–*Klebsiella pneumoniae* carbapenemase. LP = lumbar puncture. Lipo Amp = liposomal amphotericin. † death. NA = not available. ART = antiretroviral therapy: Tdf—tenofovir, Efv = efavirenz, dol—dolutegravir.

**Table 4:** Studies included in the prior and posterior distribution for Bayesian analysis regarding antigenemia prevalences in CD4 < 100 cells/µL and 100 < CD4 < 200 cells/µL groups.

Author, Year	CD4 < 100 Cells/µL				100 < CD4 < 200 Cells/µL				e Value
	*n*	Cases	Estimate	HPD Ci	*n*	Cases	Estimate	HPD Ci	
Borges_2019 [28]	159	12	0.075	[0.041; 0.123]	55	5	0.091	[0.034; 0.185]	
Deiss_2021 [31]	897	103	0.115	[0.095; 0.137]	309	14	0.045	[0.026; 0.072]	
Ezenaloue_2016 [26]	1451	52	0.036	[0.027; 0.047]	1301	12	0.009	[0.005; 0.016]	
Ferreira_2020 [27]	56	7	0.125	[0.056; 0.227]	29	3	0.103	[0.027; 0.245]	
Geda_2019 [30]	85	10	0.118	[0.061; 0.197]	43	2	0.047	[0.008; 0.139]	
Magambo_2014 [20]	73	6	0.082	[0.033; 0.160]	67	4	0.060	[0.019; 0.134]	
Mfinanga_2015 [19]	717	33	0.046	[0.032; 0.063]	268	5	0.019	[0.007; 0.040]	
Ogouyemi-Hounto_2016 [25]	155	6	0.039	[0.015; 0.077]	200	0	0.000	[0.000; 0.015]	
Rugemalila_2013 [21]	124	6	0.048	[0.019; 0.096]	94	1	0.011	[0.000; 0.049]	
Sadawadogo_2016 [24]	511	20	0.039	[0.025; 0.058]	302	7	0.023	[0.010; 0.045]	
Vidal_2016 [23]	128	4	0.031	[0.010; 0.072]	35	1	0.029	[0.001; 0.126]	
Mimicos_2021 [present study)	143	5	0.035	[0.013; 0.074]	134	3	0.022	[0.005; 0.058]	
Bayesian Analysis	-	-	0.036	[0.025; 0.057]	-	-	0.011	[0.005; 0.043]	0.307

Estimate: proportion of positive LFAs based on the posterior model; HPD Ci: highest posterior density 95% credible interval; e-value: FBST measure of evidence for comparing groups.

**Table 5:** Estimated prevalence of positive LFAs on the posterior model and HPD credible intervals for each group and FBST evidence value for group comparison.

Group	Subgroup	Estimate	HPD Ci	e-Value
CD4	0–100	0.036	[0.025; 0.057]	0.307
	100–200	0.011	[0.005; 0.043]	
Sex	Female	0.023	[0.015; 0.069]	0.975
	Male	0.025	[0.013; 0.039]	
Hospitalization	No	0.024	[0.017; 0.047]	0.896
	Yes	0.030	[0.014; 0.056]	
Antiretroviral Therapy	Regular Use	0.017	[0.004; 0.047]	0.418
	Irregular Use	0.035	[0.017; 0.061]	
Age	≤40	0.067	[0.038; 0.10]	0.387
	>40	0.039	[0.02; 0.066]	
Opportunist Disease	No	0.027	[0.011; 0.054]	0.064
	Yes	0.073	[0.045; 0.108]	
Viral Load	<100.000 copies/μL	0.063	[0.034; 0.104]	0.626
	≥100.000 copies/μL	0.043	[0.024; 0.070]	
Time Since Diagnosis	≤12 months	0.067	[0.037; 0.111]	0.499
	>12 months	0.042	[0.023; 0.069]	
Symptoms	No	0.075	[0.043; 0.119]	0.172
	Yes	0.036	[0.019; 0.061]	
Antifungal Use	No	0.033	[0.015; 0.061]	0.565
	Yes	0.000	[0.000; 0.087]	

Estimate: proportion of positive LFAs based on the posterior model; HPD Ci: highest posterior density 95% credible interval; e-value: FBST measure of evidence for comparing groups.

**Table 6:** Estimated prevalence of mortality on the posterior model and HPD credible intervals for positive and negative LFA groups and FBST evidence value for group comparison.

Mortality	Groups	Estimate	HPD Ci	e-Value
LFA	Negative LFA	0.127	[0.109; 0.147]	0.05013
	Positive LFA	0.282	[0.157; 0.434]	

Estimate: proportion of mortality based on the posterior model; HPD Ci: highest posterior density 95%. credible interval; e-value: FBST measure of evidence for comparing groups.

## Data Availability

Data will be provided as a Supplementary File.

## Supplementary Materials

The following supporting information can be downloaded at https://www.mdpi.com/article/10.3390/jof8121284/s1. Supplementary file S1: Database on Database on sociodemographic, clinical, and laboratory variables of 277 patients living with HIV/AIDS in São Paulo, Brazil.
