# Supplementary material for: Prevalence and Associated Factors of Cryptococcal Antigenemia in HIV-Infected Patients with CD4 < 200 Cells/µL in São Paulo, Brazil: A Bayesian Analysis
Source: J Fungi (Basel). 2022 Dec 8;8(12):1284. doi: 10.3390/jof8121284 (PMC9786117; doi:10.3390/jof8121284)
Supplement: Supplementary file 1 [file jof-08-01284-s001.zip › jof-1964953-supplementary.pdf]

| patient's number | region of birth | race | age | gender | sexual orientation | education | time since  diagnosis | hospitalization |
|---|---|---|---|---|---|---|---|---|
| 1 | 1 | 1 | 1 | 1 | 2 | 2 | 1 | 2 |
| 2 | 1 | 1 | 2 | 2 | 1 | 1 | 1 | 2 |
| 3 | 1 | 1 | 2 | 1 | 2 | 1 | 1 | 2 |
| 4 | 2 | 1 | 2 | 1 | 1 | 2 | 2 | 1 |
| 5 | 1 | 2 | 2 | 1 | 2 | 1 | 2 | 1 |
| 6 | 1 | 1 | 1 | 1 | 2 | 3 | 1 | 1 |
| 7 | 1 | 1 | 2 | 2 | 1 | 2 | 2 | 1 |
| 8 | 1 | 1 | 2 | 1 | 1 | 2 | 1 | 2 |
| 9 | 1 | 1 | 2 | 1 | 1 | 2 | 1 | 2 |
| 11 | 3 | 1 | 1 | 2 | 1 | 2 | 1 | 2 |
| 12 | 2 | 2 | 2 | 1 | 2 | 2 | 1 | 1 |
| 13 | 1 | 1 | 1 | 2 | 1 | 2 | 2 | 1 |
| 14 | 1 | 1 | 2 | 2 | 1 | 2 | 2 | 1 |
| 15 | 2 | 1 | 2 | 2 | 1 | 2 | 1 | 2 |
| 16 | 1 | 2 | 1 | 1 | 1 | 2 | 1 | 2 |
| 17 | 1 | 1 | 1 | 1 | 2 | 1 | 2 | 2 |
| 18 | 1 | 2 | 1 | 1 | 1 | 1 | 2 | 2 |
| 19 | 1 | 1 | 2 | 2 | 1 | 2 | 2 | 2 |
| 20 | 1 | 1 | 2 | 2 | 1 | 1 | 2 | 1 |
| 21 | 2 | 2 | 2 | 2 | 1 | 1 | 2 | 2 |
| 22 | 1 | 2 | 2 | 2 | 1 | 1 | 2 | 1 |
| 23 | 1 | 1 | 2 | 1 | 1 | 1 | 2 | 1 |
| 24 | 1 | 1 | 2 | 2 | 1 | 1 | 2 | 1 |
| 25 | 1 | 1 | 2 | 1 | 2 | 1 | 1 | 2 |
| 26 | 2 | 1 | 2 | 1 | 2 | 1 | 1 | 2 |
| 27 | 1 | 4 | 2 | 1 | 1 | 1 | 2 | 1 |
| 28 | 2 | 1 | 2 | 1 | 2 | 1 | 2 | 1 |
| 29 | 1 | 1 | 2 | 1 | 1 | 1 | 2 | 1 |
| 30 | 1 | 1 | 1 | 2 | 1 | 1 | 2 | 2 |
| 31 | 1 | 1 | 1 | 1 | 1 | 2 | 2 | 2 |

| | | | | | | | | |
|---|---|---|---|---|---|---|---|---|
| 32 | 1 | 1 | 2 | 2 | 1 | 2 | 2 | 1 |
| 33 | 1 | 1 | 2 | 2 | 1 | 1 | 2 | 1 |
| 34 | 1 | 1 | 2 | 2 | 1 | 2 | 2 | 1 |
| 35 | 1 | 1 | 2 | 1 | 2 | 2 | 2 | 1 |
| 36 | 2 | 1 | 2 | 2 | 1 | 1 | 1 | 1 |
| 37 | 3 | 1 | 2 | 2 | 1 | 1 | 2 | 1 |
| 38 | 1 | 2 | 2 | 2 | 1 | 2 | 2 | 1 |
| 39 | 2 | 1 | 1 | 1 | 1 | 1 | 2 | 2 |
| 40 | 1 | 1 | 1 | 1 | 1 | 1 | 2 | 1 |
| 41 | 2 | 1 | 2 | 1 | 1 | 1 | 2 | 1 |
| 42 | 1 | 4 | 2 | 2 | 1 | 1 | 1 | 2 |
| 44 | 3 | 1 | 1 | 2 | 1 | 3 | 2 | 2 |
| 45 | 1 | 2 | 2 | 2 | 1 | 1 | 2 | 2 |
| 46 | 2 | 1 | 1 | 2 | 1 | 1 | 2 | 1 |
| 47 | 1 | 1 | 2 | 1 | 3 | 1 | 2 | 2 |
| 48 | 1 | 1 | 2 | 2 | 1 | 1 | 2 | 1 |
| 49 | 1 | 2 | 1 | 1 | 1 | 2 | 1 | 2 |
| 50 | 2 | 2 | 2 | 1 | 1 | 1 | 2 | 2 |
| 51 | 1 | 2 | 2 | 2 | 1 | 1 | 2 | 1 |
| 52 | 1 | 1 | 1 | 1 | 2 | 2 | 1 | 2 |
| 53 | 1 | 2 | 2 | 2 | 1 | 1 | 2 | 2 |
| 54 | 1 | 1 | 1 | 1 | 2 | 1 | 2 | 2 |
| 56 | 2 | 2 | 2 | 1 | 1 | 1 | 2 | 2 |
| 57 | 1 | 1 | 2 | 2 | 1 | 1 | 2 | 1 |
| 58 | 1 | 2 | 2 | 2 | 1 | 2 | 1 | 1 |
| 59 | 1 | 2 | 2 | 1 | 2 | 2 | 2 | 1 |
| 60 | 1 | 1 | 2 | 2 | 1 | 2 | 2 | 1 |
| 61 | 1 | 1 | 2 | 2 | 1 | 2 | 2 | 1 |
| 62 | 1 | 1 | 2 | 2 | 1 | 1 | 2 | 1 |
| 63 | 1 | 2 | 2 | 1 | 2 | 2 | 2 | 2 |
| 64 | 2 | 1 | 2 | 1 | 2 | 2 | 2 | 1 |

| | | | | | | | | |
|---|---|---|---|---|---|---|---|---|
| 65 | 1 | 1 | 1 | 1 | 2 | 3 | 1 | 2 |
| 66 | 1 | 4 | 2 | 1 | 1 | 2 | 2 | 2 |
| 67 | 1 | 2 | 1 | 2 | 1 | 2 | 2 | 1 |
| 68 | 2 | 1 | 1 | 1 | 2 | 1 | 1 | 2 |
| 69 | 2 | 1 | 2 | 2 | 1 | 1 | 2 | 2 |
| 70 | 1 | 1 | 2 | 1 | 2 | 1 | 2 | 1 |
| 71 | 1 | 1 | 2 | 1 | 2 | 2 | 1 | 2 |
| 72 | 1 | 1 | 2 | 1 | 1 | 2 | 1 | 1 |
| 73 | 2 | 1 | 2 | 2 | 1 | 1 | 1 | 2 |
| 74 | 1 | 1 | 2 | 2 | 1 | 1 | 2 | 1 |
| 75 | 1 | 1 | 2 | 1 | 1 | 1 | 2 | 1 |
| 76 | 1 | 2 | 1 | 2 | 1 | 1 | 1 | 2 |
| 77 | 1 | 2 | 2 | 1 | 1 | 3 | 1 | 2 |
| 78 | 1 | 1 | 2 | 1 | 2 | 2 | 1 | 2 |
| 79 | 1 | 2 | 2 | 1 | 1 | 1 | 2 | 2 |
| 80 | 1 | 4 | 1 | 1 | 1 | 2 | 2 | 1 |
| 81 | 2 | 1 | 1 | 2 | 1 | 2 | 2 | 1 |
| 82 | 1 | 1 | 1 | 1 | 2 | 2 | 2 | 2 |
| 83 | 1 | 1 | 2 | 1 | 1 | 2 | 1 | 2 |
| 84 | 2 | 1 | 2 | 1 | 2 | 1 | 2 | 1 |
| 85 | 1 | 1 | 1 | 1 | 1 | 2 | 1 | 2 |
| 86 | 2 | 2 | 2 | 1 | 2 | 2 | 1 | 2 |
| 87 | 1 | 1 | 1 | 2 | 1 | 1 | 2 | 1 |
| 88 | 1 | 2 | 2 | 2 | 1 | 1 | 2 | 1 |
| 89 | 2 | 1 | 2 | 2 | 1 | 2 | 1 | 2 |
| 91 | 1 | 1 | 1 | 2 | 1 | 2 | 2 | 2 |
| 92 | 1 | 2 | 2 | 2 | 1 | 1 | 2 | 2 |
| 93 | 1 | 1 | 2 | 1 | 1 | 2 | 2 | 2 |
| 94 | 1 | 1 | 1 | 2 | 1 | 1 | 2 | 2 |
| 95 | 1 | 1 | 2 | 1 | 2 | 2 | 1 | 1 |
| 96 | 2 | 1 | 2 | 1 | 2 | 1 | 2 | 2 |

| | | | | | | | | |
|---|---|---|---|---|---|---|---|---|
| 97 | 2 | 2 | 2 | 1 | 2 | 1 | 2 | 1 |
| 98 | 1 | 2 | 2 | 2 | 1 | 2 | 2 | 2 |
| 99 | 1 | 1 | 1 | 2 | 1 | 1 | 2 | 2 |
| 100 | 1 | 1 | 1 | 1 | 1 | 2 | 2 | 2 |
| 101 | 1 | 2 | 1 | 1 | 3 | 1 | 2 | 2 |
| 102 | 1 | 2 | 1 | 2 | 1 | 2 | 2 | 2 |
| 103 | 1 | 1 | 2 | 1 | 1 | 2 | 2 | 2 |
| 104 | 2 | 1 | 2 | 1 | 2 | 1 | 2 | 1 |
| 105 | 1 | 1 | 2 | 1 | 2 | 2 | 1 | 2 |
| 106 | 2 | 1 | 2 | 1 | 2 | 2 | 2 | 2 |
| 107 | 1 | 2 | 2 | 1 | 2 | 1 | 2 | 1 |
| 108 | 1 | 1 | 2 | 1 | 2 | 2 | 2 | 1 |
| 109 | 1 | 1 | 2 | 1 | 1 | 1 | 1 | 2 |
| 110 | 1 | 2 | 1 | 2 | 1 | 1 | 2 | 1 |
| 111 | 1 | 1 | 2 | 1 | 1 | 1 | 2 | 1 |
| 112 | 1 | 1 | 2 | 2 | 1 | 1 | 2 | 1 |
| 113 | 3 | 1 | 1 | 1 | 1 | 2 | 1 | 2 |
| 114 | 1 | 1 | 2 | 1 | 2 | 2 | 1 | 1 |
| 115 | 2 | 1 | 1 | 2 | 1 | 1 | 2 | 2 |
| 116 | 2 | 2 | 1 | 1 | 2 | 1 | 2 | 2 |
| 117 | 1 | 1 | 1 | 1 | 1 | 1 | 2 | 1 |
| 118 | 2 | 2 | 1 | 1 | 2 | 1 | 2 | 1 |
| 119 | 1 | 4 | 1 | 1 | 2 | 2 | 1 | 2 |
| 121 | 2 | 1 | 2 | 2 | 1 | 1 | 2 | 2 |
| 122 | 1 | 2 | 2 | 1 | 1 | 1 | 2 | 2 |
| 123 | 1 | 1 | 2 | 1 | 1 | 2 | 2 | 1 |
| 124 | 1 | 1 | 1 | 2 | 1 | 1 | 2 | 2 |
| 125 | 1 | 1 | 2 | 1 | 3 | 3 | 1 | 2 |
| 126 | 3 | 1 | 2 | 2 | 2 | 2 | 2 | 2 |
| 128 | 1 | 1 | 2 | 2 | 1 | 2 | 2 | 2 |
| 129 | 1 | 2 | 2 | 2 | 1 | 1 | 2 | 2 |

| | | | | | | | | |
|---|---|---|---|---|---|---|---|---|
| 131 | 1 | 2 | 1 | 1 | 1 | 1 | 2 | 2 |
| 132 | 2 | 2 | 2 | 1 | 2 | 1 | 2 | 1 |
| 133 | 1 | 2 | 1 | 1 | 2 | 1 | 2 | 2 |
| 134 | 2 | 1 | 2 | 2 | 1 | 1 | 2 | 2 |
| 135 | 1 | 1 | 2 | 2 | 1 | 1 | 2 | 2 |
| 136 | 2 | 2 | 2 | 1 | 1 | 1 | 2 | 2 |
| 137 | 1 | 1 | 1 | 1 | 2 | 2 | 2 | 2 |
| 138 | 1 | 1 | 2 | 2 | 1 | 1 | 2 | 1 |
| 139 | 1 | 1 | 2 | 1 | 1 | 2 | 2 | 1 |
| 140 | 1 | 1 | 2 | 1 | 1 | 1 | 2 | 1 |
| 143 | 1 | 1 | 2 | 1 | 3 | 2 | 2 | 1 |
| 144 | 1 | 2 | 1 | 2 | 1 | 2 | 2 | 1 |
| 145 | 1 | 2 | 2 | 2 | 1 | 1 | 1 | 2 |
| 146 | 1 | 1 | 1 | 1 | 2 | 1 | 2 | 1 |
| 147 | 1 | 1 | 2 | 2 | 1 | 1 | 2 | 2 |
| 148 | 1 | 1 | 2 | 2 | 1 | 2 | 2 | 1 |
| 149 | 1 | 2 | 1 | 2 | 1 | 2 | 2 | 1 |
| 150 | 3 | 2 | 2 | 1 | 1 | 2 | 2 | 2 |
| 151 | 1 | 1 | 2 | 2 | 1 | 2 | 2 | 1 |
| 152 | 1 | 1 | 2 | 1 | 3 | 1 | 1 | 2 |
| 154 | 1 | 2 | 1 | 1 | 2 | 2 | 2 | 1 |
| 155 | 1 | 1 | 2 | 1 | 2 | 2 | 2 | 1 |
| 156 | 1 | 1 | 2 | 1 | 2 | 2 | 2 | 1 |
| 157 | 1 | 2 | 2 | 2 | 1 | 2 | 2 | 1 |
| 158 | 1 | 1 | 2 | 2 | 1 | 1 | 1 | 2 |
| 159 | 1 | 1 | 1 | 2 | 1 | 1 | 1 | 2 |
| 160 | 1 | 4 | 1 | 2 | 3 | 3 | 1 | 2 |
| 161 | 1 | 1 | 2 | 1 | 1 | 1 | 2 | 2 |
| 162 | 2 | 2 | 2 | 2 | 1 | 2 | 1 | 1 |
| 163 | 1 | 2 | 1 | 2 | 1 | 2 | 1 | 2 |
| 164 | 2 | 2 | 2 | 2 | 1 | 1 | 2 | 1 |

| | | | | | | | | |
|---|---|---|---|---|---|---|---|---|
| 165 | 1 | 1 | 2 | 1 | 2 | 2 | 1 | 1 |
| 166 | 1 | 1 | 2 | 1 | 1 | 3 | 2 | 2 |
| 169 | 1 | 1 | 2 | 1 | 3 | 2 | 2 | 1 |
| 170 | 1 | 2 | 1 | 1 | 3 | 2 | 2 | 1 |
| 171 | 2 | 1 | 2 | 2 | 1 | 2 | 2 | 1 |
| 172 | 2 | 1 | 2 | 2 | 3 | 3 | 1 | 2 |
| 173 | 1 | 2 | 1 | 1 | 3 | 3 | 2 | 2 |
| 174 | 1 | 1 | 1 | 2 | 3 | 1 | 2 | 2 |
| 175 | 1 | 1 | 2 | 1 | 3 | 1 | 2 | 2 |
| 176 | 2 | 1 | 1 | 1 | 3 | 2 | 2 | 2 |
| 177 | 2 | 1 | 2 | 2 | 1 | 2 | 1 | 2 |
| 178 | 1 | 1 | 1 | 2 | 1 | 2 | 2 | 2 |
| 180 | 1 | 1 | 1 | 1 | 2 | 1 | 1 | 2 |
| 181 | 1 | 1 | 1 | 2 | 1 | 3 | 2 | 2 |
| 184 | 1 | 1 | 1 | 1 | 2 | 2 | 1 | 2 |
| 185 | 2 | 1 | 2 | 1 | 1 | 1 | 1 | 2 |
| 186 | 2 | 1 | 2 | 1 | 2 | 1 | 2 | 2 |
| 187 | 3 | 1 | 2 | 2 | 1 | 2 | 1 | 2 |
| 188 | 2 | 1 | 2 | 1 | 1 | 1 | 2 | 2 |
| 189 | 1 | 1 | 2 | 2 | 1 | 2 | 2 | 2 |
| 190 | 2 | 1 | 1 | 1 | 2 | 2 | 2 | 1 |
| 191 | 1 | 2 | 1 | 2 | 1 | 2 | 2 | 2 |
| 192 | 1 | 1 | 2 | 1 | 1 | 2 | 2 | 2 |
| 193 | 1 | 1 | 2 | 1 | 1 | 2 | 2 | 2 |
| 194 | 1 | 1 | 2 | 2 | 1 | 1 | 2 | 1 |
| 195 | 1 | 1 | 2 | 1 | 1 | 1 | 2 | 2 |
| 196 | 1 | 1 | 1 | 1 | 2 | 2 | 1 | 2 |
| 197 | 2 | 2 | 1 | 1 | 2 | 2 | 1 | 2 |
| 198 | 1 | 1 | 2 | 1 | 1 | 1 | 2 | 1 |
| 199 | 1 | 1 | 1 | 1 | 2 | 2 | 1 | 1 |
| 200 | 1 | 2 | 2 | 1 | 1 | 2 | 1 | 2 |

| | | | | | | | | |
|---|---|---|---|---|---|---|---|---|
| 201 | 1 | 2 | 2 | 1 | 1 | 2 | 2 | 2 |
| 202 | 2 | 2 | 1 | 1 | 1 | 1 | 2 | 2 |
| 203 | 1 | 1 | 2 | 1 | 2 | 1 | 2 | 2 |
| 204 | 1 | 2 | 2 | 1 | 2 | 2 | 2 | 1 |
| 205 | 1 | 2 | 1 | 2 | 1 | 1 | 2 | 1 |
| 206 | 1 | 1 | 2 | 1 | 1 | 1 | 2 | 2 |
| 207 | 3 | 1 | 2 | 2 | 1 | 1 | 2 | 1 |
| 208 | 1 | 1 | 2 | 1 | 2 | 1 | 2 | 2 |
| 209 | 3 | 1 | 1 | 1 | 2 | 2 | 2 | 2 |
| 210 | 1 | 1 | 1 | 1 | 2 | 2 | 2 | 2 |
| 211 | 1 | 1 | 1 | 1 | 1 | 2 | 2 | 2 |
| 212 | 2 | 2 | 1 | 1 | 1 | 2 | 1 | 2 |
| 213 | 3 | 1 | 1 | 1 | 2 | 2 | 1 | 2 |
| 214 | 1 | 1 | 2 | 1 | 2 | 2 | 1 | 2 |
| 215 | 1 | 2 | 1 | 2 | 1 | 2 | 1 | 2 |
| 216 | 1 | 1 | 2 | 1 | 1 | 2 | 1 | 2 |
| 217 | 1 | 1 | 2 | 1 | 1 | 1 | 1 | 2 |
| 218 | 1 | 1 | 1 | 1 | 2 | 1 | 2 | 2 |
| 219 | 2 | 2 | 2 | 1 | 2 | 1 | 1 | 2 |
| 220 | 1 | 1 | 1 | 2 | 1 | 2 | 2 | 2 |
| 222 | 2 | 2 | 1 | 1 | 2 | 2 | 2 | 2 |
| 223 | 1 | 1 | 2 | 1 | 2 | 2 | 2 | 2 |
| 224 | 2 | 1 | 2 | 1 | 1 | 2 | 2 | 2 |
| 225 | 1 | 1 | 1 | 2 | 1 | 2 | 2 | 2 |
| 226 | 1 | 2 | 2 | 1 | 1 | 2 | 2 | 2 |
| 227 | 1 | 2 | 1 | 1 | 2 | 2 | 1 | 2 |
| 228 | 2 | 1 | 1 | 1 | 1 | 1 | 1 | 1 |
| 229 | 1 | 2 | 1 | 1 | 2 | 2 | 2 | 2 |
| 230 | 3 | 1 | 1 | 1 | 2 | 2 | 2 | 2 |
| 231 | 1 | 1 | 2 | 2 | 1 | 1 | 2 | 2 |
| 232 | 1 | 1 | 2 | 1 | 2 | 1 | 1 | 2 |

| | | | | | | | | |
|---|---|---|---|---|---|---|---|---|
| 233 | 2 | 1 | 2 | 1 | 1 | 1 | 2 | 2 |
| 234 | 1 | 1 | 2 | 1 | 1 | 1 | 1 | 2 |
| 235 | 2 | 2 | 2 | 1 | 1 | 1 | 1 | 1 |
| 236 | 1 | 1 | 1 | 1 | 3 | 2 | 1 | 1 |
| 237 | 1 | 1 | 2 | 1 | 2 | 1 | 2 | 2 |
| 238 | 1 | 1 | 1 | 1 | 2 | 1 | 1 | 2 |
| 239 | 1 | 1 | 2 | 1 | 2 | 1 | 1 | 2 |
| 240 | 1 | 1 | 2 | 1 | 1 | 1 | 2 | 2 |
| 241 | 3 | 1 | 2 | 1 | 1 | 2 | 1 | 2 |
| 242 | 1 | 1 | 2 | 1 | 2 | 2 | 2 | 2 |
| 243 | 1 | 1 | 1 | 1 | 1 | 1 | 2 | 2 |
| 244 | 3 | 2 | 2 | 1 | 2 | 1 | 2 | 2 |
| 246 | 1 | 1 | 2 | 2 | 1 | 1 | 2 | 2 |
| 247 | 2 | 2 | 1 | 2 | 1 | 2 | 1 | 2 |
| 248 | 1 | 2 | 1 | 1 | 2 | 2 | 2 | 1 |
| 249 | 2 | 1 | 2 | 1 | 1 | 1 | 2 | 1 |
| 250 | 2 | 1 | 2 | 1 | 2 | 1 | 2 | 2 |
| 251 | 3 | 1 | 2 | 1 | 1 | 2 | 2 | 2 |
| 252 | 1 | 2 | 2 | 1 | 2 | 2 | 2 | 1 |
| 253 | 1 | 1 | 2 | 1 | 1 | 2 | 2 | 1 |
| 254 | 2 | 3 | 2 | 1 | 2 | 2 | 2 | 1 |
| 255 | 1 | 1 | 2 | 2 | 1 | 2 | 2 | 1 |
| 256 | 1 | 2 | 2 | 1 | 1 | 1 | 2 | 1 |
| 258 | 1 | 1 | 2 | 1 | 3 | 1 | 1 | 2 |
| 263 | 1 | 2 | 1 | 2 | 1 | 2 | 1 | 2 |
| 265 | 1 | 1 | 2 | 2 | 1 | 1 | 2 | 2 |
| 266 | 1 | 1 | 2 | 1 | 1 | 2 | 2 | 1 |
| 267 | 2 | 1 | 2 | 1 | 2 | 1 | 2 | 1 |
| 269 | 1 | 1 | 2 | 1 | 1 | 2 | 2 | 3 |
| 272 | 1 | 1 | 2 | 1 | 3 | 2 | 3 | 3 |
| 273 | 1 | 2 | 1 | 1 | 2 | 2 | 2 | 1 |

| | | | | | | | | |
|---|---|---|---|---|---|---|---|---|
| 275 | 1 | 2 | 2 | 1 | 1 | 2 | 2 | 1 |
| 277 | 1 | 1 | 2 | 1 | 1 | 2 | 1 | 1 |
| 280 | 1 | 2 | 2 | 2 | 1 | 2 | 2 | 1 |
| 281 | 1 | 1 | 2 | 1 | 2 | 2 | 2 | 3 |
| 282 | 2 | 3 | 2 | 1 | 1 | 2 | 2 | 1 |
| 283 | 1 | 1 | 2 | 2 | 3 | 1 | 2 | 3 |
| 284 | 2 | 2 | 2 | 1 | 2 | 2 | 2 | 3 |
| 285 | 1 | 1 | 2 | 2 | 1 | 2 | 2 | 1 |
| 286 | 1 | 2 | 2 | 2 | 1 | 2 | 2 | 1 |
| 287 | 1 | 1 | 1 | 1 | 2 | 2 | 1 | 3 |
| 288 | 1 | 2 | 1 | 2 | 3 | 1 | 2 | 3 |
| 289 | 1 | 1 | 2 | 1 | 1 | 1 | 1 | 3 |
| 290 | 1 | 1 | 1 | 1 | 1 | 2 | 2 | 1 |
| 291 | 1 | 1 | 1 | 2 | 3 | 2 | 2 | 3 |
| 292 | 2 | 2 | 2 | 1 | 1 | 1 | 2 | 3 |
| 293 | 1 | 1 | 2 | 2 | 1 | 2 | 3 | 1 |
| 294 | 1 | 1 | 1 | 1 | 3 | 2 | 2 | 1 |
| 295 | 1 | 2 | 1 | 1 | 3 | 2 | 2 | 3 |
| 296 | 1 | 2 | 1 | 1 | 2 | 2 | 2 | 3 |
| 297 | 1 | 1 | 2 | 1 | 3 | 2 | 2 | 3 |
| 298 | 1 | 1 | 2 | 1 | 3 | 2 | 2 | 3 |
| 299 | 1 | 4 | 1 | 1 | 2 | 3 | 2 | 1 |
| 300 | 1 | 2 | 1 | 1 | 3 | 2 | 2 | 3 |
| 301 | 1 | 1 | 2 | 1 | 3 | 2 | 2 | 3 |
| 302 | 1 | 1 | 2 | 1 | 2 | 2 | 2 | 3 |
| 303 | 1 | 1 | 2 | 1 | 3 | 2 | 3 | 1 |
| 304 | 1 | 1 | 2 | 2 | 3 | 2 | 3 | 3 |
| 305 | 1 | 1 | 2 | 1 | 1 | 2 | 2 | 3 |
| 306 | 2 | 2 | 2 | 1 | 1 | 2 | 1 | 3 |
| 307 | 1 | 1 | 2 | 1 | 3 | 2 | 2 | 3 |

**Tittle:** **S1 supplementary file: Database on sociodemographic, clinical, and laboratory variables of 277 patier**

**Legend**

| **Group** | Subgroups | |
|---|---|---|
| Region of birth | Southeast | 1 |
| | Northeast | 2 |
| | Others | 3 |
| Race | White | 1 |
| | Afroamerican | 2 |
| | others | 3 |
| | Not available | 4 |
| Age | ≤ 40 years | 1 |
| | > 40 years | 2 |
| Gender | Female | 1 |
| | Male | 2 |
| Sexual Orientation | Heterosexual | 1 |
| | Homosexual | 2 |
| | Not available | 3 |
| Education | < 8 years | 1 |
| | > 9 years | 2 |
| | Not available | 3 |
| Time Since Diagnosis | ≤12 months | 1 |
| | >12 months | 2 |
| | Not available | 3 |
| Hospitalization | No | 1 |
| | Yes | 2 |
| | Not available | 3 |

| | | |
|---|---|---|
| Symptoms | No | 1 |
| | Yes | 2 |
| Opportunist Disease | No | 1 |
| | Yes | 2 |
| | Not available | 3 |
| CD4 (cells/µL) | 0-100 | 1 |
| | 100-200 | 2 |
| Viral Load (copies/µL) | < 100.000 | 1 |
| | ≥ 100.000 | 2 |
| TARV | Regular Use | 1 |
| | Irregular Use | 2 |
| Antifungal Use | No | 1 |
| | Yes | 2 |
| | Not available | 3 |
| Meningoencephalitis | No | 1 |
| | Yes | 2 |
| LFA - Lateral flow assay (serum) | Neg | 1 |
| | Pos | 2 |

| symptoms | opportunist disease | CD4 (cells/µL) | Viral Load ( copies/µL) | antiviral therapy | antifungal therapy | LFA (serum) | menigoencephalitis |
|---|---|---|---|---|---|---|---|
| 1 | 2 | 1 | 1 | 2 | 1 | **1** | 1 |
| 2 | 2 | 1 | 2 | 2 | 1 | **1** | 1 |
| 2 | 2 | 2 | 1 | 2 | 1 | **1** | 1 |
| 2 | 2 | 2 | 1 | 1 | 1 | **1** | 1 |
| 1 | 1 | 2 | 1 | 1 | 1 | **1** | 1 |
| 2 | 2 | 1 | 1 | 1 | 1 | **1** | 1 |
| 2 | 2 | 1 | 1 | 2 | 1 | **1** | 1 |
| 2 | 2 | 2 | 2 | 2 | 1 | 2 | 1 |
| 2 | 2 | 1 | 2 | 2 | 1 | **1** | 1 |
| 2 | 2 | 1 | 2 | 2 | 1 | **1** | 1 |
| 2 | 2 | 1 | 1 | 2 | 1 | **1** | 1 |
| 2 | 2 | 1 | 1 | 2 | 1 | **1** | 1 |
| 2 | 1 | 2 | 1 | 1 | 1 | **1** | 1 |
| 1 | 2 | 1 | 1 | 2 | 1 | **1** | 1 |
| 1 | 2 | 1 | 2 | 2 | 1 | **1** | 1 |
| 1 | 1 | 2 | 2 | 2 | 1 | **1** | 1 |
| 1 | 2 | 1 | 2 | 2 | 1 | **1** | 1 |
| 2 | 1 | 2 | 1 | 2 | 1 | **1** | 1 |
| 2 | 2 | 1 | 1 | 2 | 1 | **1** | 1 |
| 1 | 2 | 1 | 1 | 2 | 1 | **1** | 1 |
| 2 | 1 | 1 | 2 | 2 | 1 | **1** | 1 |
| 2 | 2 | 1 | 2 | 2 | 1 | **1** | 1 |
| 2 | 2 | 2 | 1 | 2 | 1 | **1** | 1 |
| 1 | 2 | 1 | 2 | 2 | 1 | **1** | 1 |
| 2 | 2 | 1 | 2 | 2 | 1 | **1** | 1 |
| 2 | 3 | 2 | 1 | 2 | 1 | **1** | 1 |
| 2 | 1 | 2 | 1 | 2 | 1 | **1** | 1 |
| 2 | 1 | 2 | 1 | 1 | 1 | **1** | 1 |
| 2 | 2 | 1 | 1 | 1 | 1 | **1** | 1 |
| 1 | 2 | 2 | 1 | 2 | 1 | **1** | 1 |

| | | | | | | | |
|---|---|---|---|---|---|---|---|
| 2 | 1 | 2 | 1 | 2 | 1 | **1** | 1 |
| 2 | 1 | 2 | 1 | 1 | 1 | **1** | 1 |
| 2 | 1 | 1 | 1 | 2 | 1 | **1** | 1 |
| 2 | 1 | 2 | 1 | 1 | 1 | **1** | 1 |
| 2 | 1 | 2 | 1 | 1 | 1 | **1** | 1 |
| 2 | 1 | 2 | 1 | 1 | 1 | **1** | 1 |
| 2 | 2 | 1 | 1 | 2 | 1 | **1** | 1 |
| 1 | 2 | 1 | 2 | 2 | 1 | **1** | 1 |
| 2 | 1 | 1 | 1 | 2 | 1 | **1** | 1 |
| 2 | 1 | 1 | 1 | 2 | 1 | **1** | 1 |
| 1 | 2 | 1 | 1 | 1 | 1 | **1** | 1 |
| 1 | 1 | 1 | 1 | 2 | 1 | **1** | 1 |
| 2 | 2 | 2 | 2 | 2 | 1 | **1** | 1 |
| 2 | 2 | 1 | 1 | 2 | 1 | 2 | 1 |
| 1 | 1 | 1 | 2 | 2 | 1 | **1** | 1 |
| 2 | 1 | 2 | 1 | 2 | 1 | **1** | 1 |
| 1 | 2 | 2 | 1 | 2 | 1 | **1** | 1 |
| 1 | 1 | 1 | 1 | 1 | 3 | **1** | 1 |
| 2 | 1 | 2 | 1 | 2 | 1 | **1** | 1 |
| 1 | 2 | 1 | 1 | 2 | 2 | **1** | 1 |
| 1 | 2 | 1 | 2 | 2 | 1 | **1** | 1 |
| 1 | 2 | 2 | 1 | 2 | 1 | **1** | 1 |
| 1 | 2 | 1 | 2 | 2 | 1 | **1** | 1 |
| 2 | 1 | 1 | 1 | 1 | 1 | **1** | 1 |
| 2 | 1 | 2 | 1 | 1 | 1 | 2 | 1 |
| 2 | 1 | 1 | 1 | 2 | 1 | **1** | 1 |
| 2 | 3 | 2 | 1 | 2 | 1 | **1** | 1 |
| 2 | 1 | 2 | 1 | 1 | 1 | **1** | 1 |
| 1 | 2 | 1 | 2 | 2 | 1 | **1** | 1 |
| 1 | 2 | 1 | 1 | 1 | 2 | **1** | 1 |
| 2 | 1 | 1 | 1 | 1 | 1 | **1** | 1 |

| | | | | | | | |
|---|---|---|---|---|---|---|---|
| 1 | 2 | 1 | 2 | 2 | 1 | **1** | 1 |
| 2 | 2 | 2 | 1 | 1 | 1 | **1** | 1 |
| 2 | 2 | 2 | 1 | 2 | 1 | **1** | 1 |
| 1 | 2 | 1 | 2 | 2 | 1 | **1** | 1 |
| 1 | 1 | 1 | 2 | 2 | 1 | **1** | 1 |
| 2 | 1 | 2 | 1 | 1 | 1 | **1** | 1 |
| 1 | 2 | 2 | 2 | 2 | 1 | **1** | 1 |
| 2 | 1 | 1 | 1 | 2 | 1 | **1** | 1 |
| 1 | 1 | 1 | 2 | 2 | 1 | **1** | 1 |
| 2 | 1 | 2 | 1 | 1 | 1 | **1** | 1 |
| 2 | 1 | 1 | 1 | 1 | 1 | **1** | 1 |
| 1 | 2 | 1 | 1 | 2 | 1 | 2 | 2 |
| 1 | 2 | 1 | 1 | 2 | 1 | **1** | 1 |
| 1 | 3 | 1 | 2 | 2 | 1 | **1** | 1 |
| 2 | 1 | 2 | 1 | 1 | 1 | **1** | 1 |
| 2 | 1 | 1 | 1 | 2 | 1 | **1** | 1 |
| 2 | 1 | 1 | 1 | 2 | 1 | **1** | 1 |
| 1 | 2 | 1 | 2 | 2 | 1 | **1** | 1 |
| 1 | 1 | 1 | 2 | 2 | 1 | **1** | 1 |
| 2 | 1 | 1 | 2 | 2 | 1 | **1** | 1 |
| 1 | 2 | 1 | 1 | 2 | 1 | **1** | 1 |
| 2 | 3 | 1 | 2 | 2 | 1 | **1** | 1 |
| 2 | 1 | 1 | 1 | 2 | 1 | **1** | 1 |
| 2 | 1 | 1 | 1 | 2 | 1 | **1** | 1 |
| 2 | 2 | 1 | 2 | 2 | 2 | **1** | 1 |
| 1 | 3 | 2 | 1 | 1 | 1 | **1** | 1 |
| 1 | 2 | 1 | 1 | 2 | 2 | **1** | 1 |
| 1 | 2 | 1 | 2 | 2 | 1 | **1** | 1 |
| 1 | 2 | 2 | 2 | 2 | 2 | **1** | 1 |
| 2 | 1 | 2 | 1 | 2 | 1 | **1** | 1 |
| 1 | 2 | 1 | 2 | 2 | 1 | **1** | 1 |

| | | | | | | | |
|---|---|---|---|---|---|---|---|
| 2 | 2 | 1 | 1 | 2 | 1 | **1** | 1 |
| 1 | 2 | 2 | 2 | 2 | 1 | **1** | 1 |
| 2 | 2 | 1 | 2 | 2 | 1 | **1** | 1 |
| 1 | 2 | 1 | 2 | 2 | 1 | **1** | 1 |
| 2 | 3 | 1 | 1 | 2 | 1 | **1** | 1 |
| 2 | 2 | 2 | 1 | 1 | 1 | **1** | 1 |
| 1 | 2 | 1 | 2 | 2 | 1 | **1** | 1 |
| 2 | 2 | 1 | 1 | 2 | 2 | **1** | 1 |
| 2 | 1 | 2 | 1 | 2 | 1 | **1** | 1 |
| 1 | 1 | 1 | 2 | 2 | 1 | **1** | 1 |
| 2 | 1 | 2 | 1 | 1 | 1 | **1** | 1 |
| 2 | 2 | 1 | 1 | 2 | 1 | **1** | 1 |
| 1 | 2 | 1 | 1 | 2 | 1 | **1** | 1 |
| 2 | 1 | 2 | 1 | 1 | 1 | **1** | 1 |
| 2 | 1 | 2 | 1 | 1 | 1 | **1** | 1 |
| 2 | 1 | 2 | 1 | 2 | 1 | **1** | 1 |
| 1 | 2 | 1 | 2 | 2 | 1 | **1** | 1 |
| 2 | 1 | 1 | 1 | 2 | 1 | **1** | 1 |
| 1 | 2 | 2 | 1 | 2 | 1 | **1** | 1 |
| 1 | 2 | 1 | 1 | 2 | 2 | **1** | 1 |
| 2 | 1 | 1 | 1 | 2 | 1 | **1** | 1 |
| 2 | 3 | 1 | 1 | 1 | 1 | **1** | 1 |
| 1 | 3 | 1 | 1 | 2 | 1 | 1 | 1 |
| 1 | 2 | 1 | 2 | 2 | 1 | 1 | 1 |
| 1 | 1 | 2 | 1 | 1 | 1 | **1** | 1 |
| 2 | 1 | 2 | 1 | 1 | 1 | **1** | 1 |
| 1 | 2 | 1 | 2 | 2 | 1 | **1** | 1 |
| 2 | 1 | 1 | 1 | 2 | 1 | **1** | 1 |
| 2 | 2 | 1 | 1 | 1 | 1 | **1** | 1 |
| 1 | 2 | 1 | 2 | 2 | 1 | **1** | 1 |
| 1 | 3 | 1 | 1 | 2 | 1 | **1** | 1 |

| | | | | | | | |
|---|---|---|---|---|---|---|---|
| 2 | 2 | 2 | 1 | 2 | 1 | **1** | 1 |
| 1 | 2 | 1 | 1 | 2 | 1 | **1** | 1 |
| 2 | 2 | 1 | 2 | 2 | 2 | **1** | 1 |
| 1 | 2 | 1 | 1 | 2 | 2 | **1** | 1 |
| 2 | 2 | 1 | 2 | 2 | 1 | **1** | 1 |
| 1 | 2 | 1 | 1 | 2 | 1 | **1** | 1 |
| 2 | 2 | 2 | 2 | 2 | 1 | **1** | 1 |
| 1 | 2 | 1 | 1 | 2 | 1 | **1** | 1 |
| 2 | 1 | 2 | 1 | 1 | 1 | 2 | 1 |
| 2 | 2 | 2 | 1 | 2 | 1 | 1 | 1 |
| 1 | 2 | 2 | 1 | 2 | 1 | 1 | 1 |
| 2 | 2 | 2 | 1 | 1 | 1 | 1 | 1 |
| 1 | 2 | 1 | 2 | 2 | 2 | 1 | 1 |
| 2 | 1 | 2 | 1 | 2 | 1 | 1 | 1 |
| 2 | 2 | 1 | 1 | 2 | 2 | 1 | 1 |
| 1 | 2 | 1 | 1 | 1 | 1 | 1 | 1 |
| 1 | 2 | 2 | 1 | 2 | 1 | 1 | 1 |
| 2 | 1 | 1 | 2 | 2 | 1 | 1 | 1 |
| 2 | 1 | 2 | 1 | 1 | 1 | **1** | 1 |
| 2 | 2 | 1 | 2 | 2 | 1 | **1** | 1 |
| 2 | 2 | 2 | 1 | 1 | 1 | 1 | 1 |
| 2 | 2 | 1 | 1 | 1 | 1 | 1 | 1 |
| 2 | 1 | 2 | 1 | 2 | 1 | 1 | 1 |
| 2 | 1 | 1 | 1 | 2 | 1 | 1 | 1 |
| 1 | 2 | 1 | 2 | 2 | 2 | 1 | 1 |
| 2 | 2 | 1 | 2 | 2 | 1 | 1 | 1 |
| 1 | 2 | 1 | 2 | 2 | 1 | 1 | 1 |
| 2 | 1 | 2 | 1 | 2 | 1 | 1 | 1 |
| 1 | 2 | 2 | 1 | 1 | 1 | 1 | 1 |
| 1 | 1 | 1 | 1 | 2 | 1 | 2 | 2 |
| 2 | 1 | 1 | 2 | 2 | 1 | 1 | 1 |

| | | | | | | | |
|---|---|---|---|---|---|---|---|
| 1 | 2 | 2 | 1 | 1 | 1 | 1 | 1 |
| 1 | 2 | 1 | 2 | 2 | 1 | 1 | 1 |
| 1 | 2 | 2 | 1 | 1 | 2 | 1 | 1 |
| 1 | 2 | 1 | 1 | 2 | 1 | 1 | 1 |
| 2 | 1 | 2 | 1 | 2 | 1 | **1** | 1 |
| 2 | 2 | 1 | 1 | 2 | 1 | 2 | 1 |
| 2 | 1 | 1 | 1 | 2 | 1 | 1 | 1 |
| 2 | 2 | 1 | 2 | 2 | 1 | 1 | 1 |
| 2 | 2 | 2 | 1 | 2 | 1 | 1 | 1 |
| 2 | 2 | 1 | 1 | 2 | 1 | 1 | 1 |
| 1 | 2 | 2 | 1 | 2 | 2 | 1 | 1 |
| 2 | 2 | 1 | 2 | 2 | 3 | **1** | 1 |
| 1 | 2 | 1 | 1 | 2 | 2 | 1 | 1 |
| 1 | 1 | 2 | 2 | 2 | 2 | 1 | 1 |
| 1 | 1 | 2 | 1 | 2 | 1 | 1 | 1 |
| 1 | 2 | 1 | 2 | 2 | 1 | 1 | 1 |
| 1 | 1 | 1 | 2 | 2 | 2 | 1 | 1 |
| 1 | 2 | 1 | 2 | 2 | 1 | 1 | 1 |
| 1 | 2 | 1 | 1 | 1 | 1 | 1 | 1 |
| 1 | 2 | 1 | 2 | 2 | 1 | 1 | 1 |
| 1 | 2 | 2 | 2 | 2 | 1 | 1 | 1 |
| 1 | 2 | 1 | 2 | 2 | 1 | 1 | 1 |
| 2 | 1 | 2 | 1 | 1 | 1 | 1 | 1 |
| 2 | 2 | 1 | 2 | 2 | 1 | 1 | 1 |
| 1 | 2 | 1 | 1 | 1 | 1 | 1 | 1 |
| 1 | 2 | 1 | 1 | 2 | 1 | 1 | 1 |
| 1 | 2 | 1 | 2 | 2 | 2 | 1 | 1 |
| 1 | 2 | 1 | 1 | 2 | 1 | 1 | 1 |
| 1 | 2 | 2 | 1 | 2 | 2 | 1 | 1 |
| 1 | 2 | 2 | 1 | 2 | 1 | 1 | 1 |
| 1 | 2 | 1 | 1 | 2 | 1 | 1 | 1 |

| | | | | | | | |
|---|---|---|---|---|---|---|---|
| 1 | 2 | 2 | 1 | 2 | 1 | 1 | 1 |
| 1 | 2 | 1 | 1 | 2 | 1 | 1 | 1 |
| 1 | 1 | 2 | 1 | 2 | 1 | 1 | 1 |
| 2 | 1 | 1 | 1 | 1 | 1 | 1 | 1 |
| 1 | 2 | 1 | 1 | 2 | 1 | 1 | 1 |
| 1 | 1 | 2 | 1 | 2 | 2 | 1 | 1 |
| 1 | 1 | 2 | 1 | 1 | 1 | 1 | 1 |
| 1 | 2 | 2 | 1 | 2 | 1 | 1 | 1 |
| 1 | 2 | 1 | 1 | 2 | 2 | 1 | 1 |
| 1 | 2 | 1 | 1 | 1 | 1 | 1 | 1 |
| 1 | 2 | 1 | 2 | 2 | 1 | 1 | 1 |
| 1 | 1 | 2 | 2 | 2 | 1 | 1 | 1 |
| 1 | 2 | 2 | 1 | 2 | 1 | 1 | 1 |
| 1 | 2 | 1 | 2 | 2 | 1 | 1 | 1 |
| 1 | 2 | 1 | 2 | 2 | 2 | 1 | 1 |
| 1 | 1 | 1 | 2 | 2 | 1 | 1 | 1 |
| 1 | 3 | 1 | 2 | 2 | 1 | 1 | 1 |
| 1 | 2 | 1 | 1 | 1 | 1 | 1 | 1 |
| 1 | 2 | 1 | 2 | 2 | 1 | 1 | 1 |
| 1 | 2 | 1 | 2 | 2 | 1 | 1 | 1 |
| 1 | 2 | 1 | 1 | 2 | 1 | 1 | 1 |
| 1 | 2 | 2 | 1 | 2 | 2 | 1 | 1 |
| 1 | 2 | 1 | 1 | 2 | 1 | 1 | 1 |
| 1 | 1 | 2 | 1 | 2 | 1 | 1 | 1 |
| 1 | 2 | 1 | 1 | 2 | 1 | 1 | 1 |
| 1 | 2 | 2 | 1 | 2 | 1 | 1 | 1 |
| 1 | 2 | 1 | 1 | 2 | 2 | 1 | 1 |
| 1 | 2 | 1 | 2 | 2 | 1 | 1 | 1 |
| 1 | 2 | 2 | 1 | 2 | 1 | 1 | 1 |
| 1 | 2 | 2 | 1 | 1 | 1 | 1 | 1 |
| 1 | 2 | 1 | 1 | 2 | 1 | 1 | 1 |

| | | | | | | | |
|---|---|---|---|---|---|---|---|
| 1 | 1 | 2 | 2 | 2 | 1 | 1 | 1 |
| 1 | 1 | 1 | 1 | 2 | 1 | 1 | 1 |
| 1 | 2 | 1 | 1 | 2 | 1 | 1 | 1 |
| 1 | 2 | 2 | 1 | 2 | 1 | 1 | 1 |
| 2 | 1 | 2 | 1 | 2 | 1 | 1 | 1 |
| 2 | 2 | 2 | 2 | 2 | 1 | 1 | 1 |
| 1 | 2 | 1 | 2 | 2 | 1 | 2 | 1 |
| 2 | 2 | 1 | 2 | 2 | 1 | 1 | 1 |
| 1 | 2 | 2 | 2 | 2 | 1 | 1 | 1 |
| 1 | 1 | 2 | 1 | 2 | 1 | 1 | 1 |
| 1 | 2 | 2 | 1 | 2 | 2 | 1 | 1 |
| 1 | 1 | 2 | 2 | 2 | 1 | 1 | 1 |
| 1 | 2 | 1 | 2 | 2 | 2 | 1 | 1 |
| 1 | 2 | 2 | 2 | 2 | 2 | 1 | 1 |
| 2 | 2 | 2 | 2 | 2 | 1 | 1 | 1 |
| 2 | 1 | 2 | 1 | 2 | 1 | 1 | 1 |
| 1 | 2 | 2 | 2 | 2 | 1 | 1 | 1 |
| 1 | 2 | 2 | 2 | 2 | 1 | 1 | 1 |
| 2 | 1 | 2 | 1 | 2 | 1 | 1 | 1 |
| 2 | 2 | 2 | 1 | 2 | 1 | 1 | 1 |
| 2 | 2 | 2 | 1 | 1 | 1 | 1 | 1 |
| 2 | 1 | 2 | 1 | 1 | 1 | 1 | 1 |
| 2 | 1 | 2 | 1 | 1 | 2 | 1 | 1 |
| 1 | 2 | 1 | 2 | 2 | 1 | 1 | 1 |
| 1 | 2 | 1 | 2 | 2 | 2 | 1 | 1 |
| 1 | 1 | 2 | 2 | 2 | 2 | 1 | 1 |
| 2 | 1 | 2 | 1 | 1 | 2 | 1 | 1 |
| 2 | 3 | 2 | 1 | 1 | 1 | 1 | 1 |
| 2 | 1 | 2 | 1 | 1 | 1 | 1 | 1 |
| 2 | 2 | 2 | 1 | 2 | 1 | 1 | 1 |
| 2 | 1 | 2 | 1 | 1 | 1 | 1 | 1 |

| | | | | | | | |
|---|---|---|---|---|---|---|---|
| 2 | 1 | 2 | 1 | 1 | 1 | 1 | 1 |
| 1 | 2 | 2 | 1 | 2 | 1 | 1 | 1 |
| 2 | 1 | 2 | 1 | 1 | 1 | 1 | 1 |
| 2 | 1 | 2 | 1 | 1 | 1 | **1** | 1 |
| 2 | 1 | 2 | 1 | 2 | 1 | 1 | 1 |
| 2 | 1 | 2 | 1 | 2 | 1 | 1 | 1 |
| 2 | 1 | 2 | 1 | 2 | 1 | 1 | 1 |
| 2 | 2 | 2 | 1 | 2 | 1 | 1 | 1 |
| 2 | 1 | 2 | 1 | 2 | 1 | 1 | 1 |
| 2 | 2 | 2 | 2 | 2 | 1 | 1 | 1 |
| 2 | 1 | 2 | 1 | 1 | 1 | 1 | 1 |
| 2 | 2 | 2 | 1 | 2 | 1 | 1 | 1 |
| 2 | 1 | 2 | 1 | 1 | 1 | 1 | 1 |
| 2 | 2 | 2 | 1 | 2 | 1 | 1 | 1 |
| 2 | 2 | 2 | 1 | 1 | 1 | 1 | 1 |
| 2 | 1 | 2 | 1 | 1 | 1 | 1 | 1 |
| 2 | 1 | 2 | 1 | 2 | 1 | 1 | 1 |
| 2 | 1 | 2 | 1 | 2 | 1 | 1 | 1 |
| 2 | 2 | 2 | 1 | 1 | 1 | 1 | 1 |
| 2 | 2 | 2 | 1 | 1 | 2 | 1 | 1 |
| 2 | 1 | 2 | 1 | 1 | 1 | 1 | 1 |
| 2 | 1 | 2 | 1 | 1 | 1 | 1 | 1 |
| 2 | 1 | 2 | 1 | 1 | 1 | 1 | 1 |
| 2 | 2 | 2 | 1 | 2 | 1 | 1 | 1 |
| 2 | 1 | 2 | 1 | 1 | 1 | 1 | 1 |
| 2 | 3 | 2 | 1 | 2 | 1 | 1 | 1 |
| 2 | 1 | 2 | 1 | 1 | 1 | 1 | 1 |
| 2 | 1 | 2 | 1 | 2 | 1 | 1 | 1 |
| 1 | 2 | 2 | 1 | 1 | 1 | 1 | 1 |
| 2 | 1 | 2 | 1 | 1 | 1 | 1 | 1 |
